# *In Vitro* Activity of 2-methoxy-1,4-naphthoquinone and Stigmasta-7,22-diene-3**β**-ol from *Impatiens balsamina* L. against Multiple Antibiotic-Resistant *Helicobacter pylori*

**DOI:** 10.1093/ecam/nep147

**Published:** 2011-02-14

**Authors:** Yuan-Chuen Wang, Wan-Yu Li, Deng-Chyang Wu, Jeh-Jeng Wang, Cheng-Hsun Wu, Jyun-Ji Liao, Cheng-Kun Lin

**Affiliations:** ^1^Department of Food Science and Biotechnology, National Chung Hsing University, Taichung, Taiwan; ^2^Division of Gastroenterology, Department of Internal Medicine, Kaoshing Medical University, Kaohsiung, Taiwan; ^3^Graduate Institute of Healthcare Administration, College of Health Sciences, Kaoshing Medical University, Kaohsiung, Taiwan; ^4^Faculty of Medicinal and Applied Chemistry, Kaoshing Medical University, Kaohsiung, Taiwan; ^5^Department of Chemistry, National Chung Hsing University, Taichung, Taiwan

## Abstract

Infection with *Helicobacter pylori* is strongly associated with gastric cancer and gastric adenocarcinoma. WHO classified *H. pylori* as a group 1 carcinogen in 1994. *Impatiens balsamina* L. has been used as indigenous medicine in Asia for the treatment of rheumatism, fractures and fingernail inflammation. In this study, we isolated anti-*H. pylori* compounds from this plant and investigated their anti- and bactericidal activity. Compounds of 2-methoxy-1,4-naphthoquinone (MeONQ) and stigmasta-7,22-diene-3**β**-ol (spinasterol) were isolated from the pods and roots/stems/leaves of *I. balsamina* L., respectively. The minimum inhibitory concentrations (MICs) and minimum bactericidal concentrations (MBCs) for MeONQ were in the ranges of 0.156–0.625 and 0.313–0.625 **μ**g mL^−1^, respectively, and in the ranges of 20–80 **μ**g mL^−1^ both of MICs and MBCs for spinasterol against antibiotic (clarithromycin, metronidazole and levofloxacin) resistant *H. pylori*. Notably, the activity of MeONQ was equivalent to that of amoxicillin (AMX). The bactericidal *H. pylori* action of MeONQ was dose-dependent. Furthermore, the activity of MeONQ was not influenced by the environmental pH values (4–8) and demonstrated good thermal (121°C for 15 min) stability. MeONQ abounds in the *I. balsamina* L. pod at the level of 4.39% (w/w db). In conclusion, MeONQ exhibits strong potential to be developed as a candidate agent for the eradication of *H. pylori* infection.

## 1. Introduction

Infection with *Helicobacter pylori* is strongly associated with gastric cancer and gastric adenocarcinoma [1]. WHO classified *H. pylori* as a group 1 carcinogen in 1994. Antibiotics combined with proton-pump inhibitors, H_2_-blockers and bismuth salts are the suggested standard treatment modalities to eradicate *H. pylori* [2]. However, *H. pylori* resistance to antibiotics is thought to be the most important factor in eradication failure. Resistance rates are the highest for metronidazole (MTZ) (19.9–39.2%), followed by clarithromycin (CLR) (1.7–27.7%), and the lowest for amoxicillin (AMX), tetracycline and trovafloxacin (0–4.7%) [3, 4].

*Impatiens balsamina* L. (Balsaminaceae) is an annual herb which originated in Asia. The whole plant has been used as indigenous medicine in Taiwan for the treatment of rheumatism, swelling and fingernail inflammation. Modern pharmacological studies have reported this plant demonstrating antifungal, antibacterial, antitumor, antipruritic and antianaphylactic activities [5, 6]. The active compounds isolated from this plant include peptides (Ib-AMP1-4) from seeds, quinones[1, 4-naphthoquinone, lawsone, 2-methoxy-1,4-naphthoquinone (MeONQ), balsaquinone, impatienol, naphthalene-1,4-dione] from petals, pericarp and aerial parts, and flavonoids (kaempferol, quercetin, rutin, astragalin, nicotiflorin, naringenin and their derivatives) from petals and leaves [5–8].

**Figure 1:**
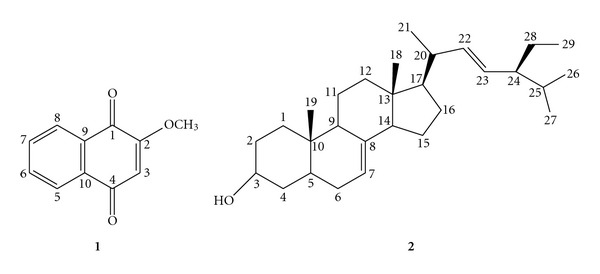
Chemical structures of compounds (**1**) and (**2**) from *I. balsamina* L.: (**1**) 2-methoxy-1,4-naphthoquinone (MeONQ); (**2**) and stigmasta-7,22-diene-3*β*-ol (spinasterol).

MeONQ (Figure 1) was isolated from the genus of *Impatiens* including *I. balsamina* L., *I. bicolor* and *I. glandulifera* Royl [7–10]. The plant *Swertia calycina* (Gentianaceae) was also found to contain this compound [11]. Some bioactivities were demonstrated in MeONQ including antipruritic [12], antiinflammatory, antiallergic [13] and anticancer [14] activities. Furthermore, reducing oral lesion recurrence in human immunodeficiency virus patients was also reported for this compound [15]. Antimicrobial activity against fungal and bacterial pathogens were also published [7, 16].

Spinasterol (Figure 1) was isolated from the plants of *I. balsamina*, *Adenophora tetraphylla*, *Medicago sativa*, *Polyalthia cerasoides* Bedd and *Pueraria mirifica* [8, 17–20]. The bioactivity reported in spinasterol includes antibacterial action against *Streptococcus mutans* and *S. sorbrinus* [19] and antitumor effect against breast, ovarian and skin cancer cells [20].

In our previous study, we revealed the whole plant of *I. balsamina* L. exhibiting strong bactericidal *H. pylori* activity, especially, the acetone and ethyl acetate pod extracts. This activity was higher than any previously reported natural products [21].

Therefore, in the current study, we isolated anti-*H. pylori* compounds from *I. balsamina* L., in which a silica gel chromatography was used. Minimum inhibiory concentrations (MICs), minimum bactericidal concentrations (MBCs), time-kill assay, effect of environmental pH, and thermal stability of the isolated compounds were examined. Multiple antibiotic-resistant *H. pylori* strains were used in the tests.

## 2. Methods

### 2.1. Plant Materials

*Impatiens balsamina* L. (white flower) were collected from a local farm in Erhshuei township between May 2007 and August 2007, which were identified by Professor Yang-Shiun Chang from the Institute of the Chinese Pharmaceutical Science China Medical University. Voucher specimens (No. 258526) were deposited at the Institute of Ecology and Evolutionary Biology, College of Life Science, National Taiwan University. The parts of pods and mixed roots/stems/leaves were dried by a warm stream of air (below 60°C) and ground (0.25 mm particle size) for the anti-*H. pylori* compounds isolation.

### 2.2. Extraction and Isolation

The *I. balsamina* L. pod extract was prepared according to Wang and Huang [22]. In brief, a mixture of 100 g of pod sample and 700 mL of acetone was stirred at room temperature for 1 h, and then was centrifuged at 13,666 g for 15 min at 4°C. The residue was extracted twice more with 700 mL of acetone of each time. All the supernatants were collected and concentrated in a rotary vacuum evaporator at less than 40°C, and thus the pod extract (7.28 g) was obtained. This extract was subjected to a silica gel column [Kiesel gel 60 (40–63 *μ*m), Merck, Germany; 2.5 cm i.d. × 33 cm] chromatography eluted with dichloromethane-methanol (150 : 1, v/v) to develop two bands in the column. The upper band was collected and subjected to the second silica gel column chromatography eluted with ethyl acetate-dichloromethane (1 : 1); one band was developed in the column and was collected. The collected fraction was subjected to the third silica gel column chromatography orderly eluted with *n*-hexane-ethyl acetate (10 : 0, 9 : 1, 8 : 2). From the 8 : 2 elution, one band was developed in the column and was collected to yield compound (**1**) (2.61 g).

Samples (100 g) of roots/stems/leaves were extracted with 95% ethanol according to the aforementioned method and 13.11 g of extract were obtained. This extract was fractionated with water and *n*-hexane. The *n*-hexane fraction was collected (1.88 g) and subjected to a silica gel column chromatography orderly eluted with *n*-hexane-ethyl acetate (10 : 0, 9 : 1 and 8 : 2) to yield two bands in the column from the 8 : 2 elution. The lower band was collected (0.638 g) and then subjected to the second silica gel column chromatography orderly eluted with *n*-hexane–ethyl acetate (100 : 0, 95 : 5, 90 : 10, 85 : 15). From the 85 : 15 elution, two bands were developed in the column. The upper band was collected and subjected to the third silica gel column chromatography eluted with *n*-hexane–ethyl acetate (75 : 25) to give three bands in the column. The middle band was collected to yield compound (**2**) (0.089 g).

### 2.3. Instrumentations

UV and IR spectra were recorded on a UV/Visible Hitachi U-2800 (Japan) spectrophotometer and a Bruker Equinox 55 (USA) FTIR, respectively. ^1^H and ^13^C NMR spectra were recorded at 600 and 150 MHz, respectively, on a Varian Inova 600 (USA) spectrometer. Chemical shifts are reported in *δ* units (ppm) and coupling constants (*J*) in H_Z_. CDCl_3_ (Sigma-Aldrich, St. Louis, MO, USA) was used as the solvent and TMS (Sigma-Aldrich, St. Louis, MO, USA) as the internal standard, in which chemical shifts for CDCl_3_ were *δ*_H_ 7.266 and *δ*_C_ 77.335. MS spectra were measured on a EI-Finnigan/Thermo Quest MAT 95 XL (Germany) mass spectrometer. Melting point was performed on the Mel-Temp (USA).

### 2.4. Bacterial Strains and Cultivation

*Helicobacter pylori* strains of ATCC 700824, 43504, and 43526 were obtained from American Type Culture Collection (ATCC). *Helicobacter pylori* strains of KMUH 4917, 4952, and 4967 were isolated from the stomach of patients from Chung-Ho Memorial Hospital, Kaohsiung Medical University. Of these *H. pylori* strains, the three ATCC strains have resistance to MTZ, the three clinical strains are cross-resistance to CLR, MTZ and levofloxacin (LVX); and all the test strains are sensitive to AMX.

A volume of 0.1 mL of *H. pylori* suspension was added in 5 mL tryptic soy broth (TSB, Difco, USA, pH 7.3), with Columbia agar (bioMérieux, France, pH 7.3) slant containing 5% (v/v) defibrinated sheep blood formed at the bottom of the test tube. The broth was incubated in a microaerophilic jar system (BBL, USA; 5% O_2_ and 10% CO_2_ in air, an OXOID BR 056A gas-generating kit was used) at 37°C for 72 h to produce 0.5–2.0 × 10^7^ cfu mL^−1^ of the bacterial counts.

### 2.5. MIC and MBC Testing

The MICs and MBCs were determined using an agar-dilution method according to Wang and Huang [22]. The test compound was dissolved in dimethyl sulfoxide (DMSO) and 2-fold diluted in Columbia agar containing 5% (v/v) defibrinated sheep blood. A volume of 0.1 mL of *H. pylori* suspension (0.5–2.0 × 10^7^ cfu mL^−1^) was spread onto a Columbia agar plate containing 5% (v/v) defibrinated sheep blood. After incubation in a microaerophilic jar system (5% O_2_ and 10% CO_2_ in air) at 37°C for 72 h, the colonies formed on the plate were enumerated. AMX and MTZ (Sigma-Aldrich, St. Louis, MO, USA) were used as the positive controls. Experiments were performed in triplicate. The MIC was defined as the lowest concentration of the test sample at which no colonies of the test *H. pylori* on the plate were formed.

For MBC testing, the test compound was dissolved in DMSO and 2-fold diluted in Columbia agar containing 5% (v/v) defibrinated sheep blood or TSB, and was then individually added to test tubes, with Columbia agar slant formed at the bottom of the test tube and the TSB layer to be covered on the slant, both the slant and broth containing the same concentrations of the test compound. *Helicobacter pylori* suspensions were added to the TSB layer to produce 2–8 × 10^5^ cfu mL^−1^ of the initial bacterial counts. The suspension was then incubated in a microaerophilic jar system (5% O_2_ and 10% CO_2_ in air) at 37°C for 72 h. A volume of 0.1 mL of each suspension was spread onto a Columbia agar plate containing 5% (v/v) defibrinated sheep blood, without the test compound. After being incubated in a microaerophilic jar system (5% O_2_ and 10% CO_2_ in air) at 37°C for another 72 h, the colonies formed were subsequently enumerated. AMX and MTZ were used as the positive controls. Experiments were performed in triplicate. The MBC was defined as the lowest concentration of the test sample giving complete inhibition of colony formation of the test *H. pylori* at the latter cultivation.

### 2.6. Time-Kill Assay of MeONQ

Forty milliliters of Columbia agar containing 5% (v/v) defibrinated sheep blood and 0–2.5 *μ*g mL^−1^ (control, 1, 2 and 4 times MBC) MeONQ were added to a 250 mL flask to create a plate at the bottom of the flask. Fifty milliliters of TSB containing the same concentrations of MeONQ were poured onto the plate. A volume of 1 mL of the *H. pylori* ATCC 700824 suspension was added to the TSB layer to produce the initial bacterial concentrations of 2–8 × 10^5^ cfu mL^−1^. The broth was incubated in a microaerophilic jar system (5% O_2_ and 10% CO_2_ in air) at 37°C for 72 h. At various intervals during the incubation period, the broths were taken and diluted by 10-fold in 0.1% peptone solution. A volume of 0.1 mL of each suspension was spread onto Columbia agar plate containing 5% (v/v) defibrinated sheep blood, without MeONQ. After incubation in a microaerophilic jar system (5% O_2_ and 10% CO_2_ in air) at 37°C for 72 h, the colonies formed on the plate were enumerated. Experiments were performed in triplicate.

### 2.7. Effect of pH of MeONQ

The effects of pH of MeONQ on the bactericidal activity against *H. pylori* was determined. Five milliliters of the Columbia agar containing 5% (v/v) defibrinated sheep blood at the final concentrations of 0–1.25 *μ*g mL^−1^ (control, MIC, and 1 and 2 times MBC) of MeONQ was added to test tubes to create slants at the bottom. Four milliliters of TSB (adjusted to pH 2–8 with 3 N HCl or 3 N NaOH) containing the same concentrations of MeONQ was poured onto the slants. A volume of 0.1 mL of the *H. pylori* ATCC 700824 suspension was added to the TSB layer to produce the initial bacterial concentrations of 2–8 × 10^5^ cfu mL^−1^. The broth was incubated in a microaerophilic jar system (5% O_2_ and 10% CO_2_ in air) at 37°C for 24 h. Each of the broth was sampled and diluted by 10-fold in 0.1% peptone solution. A volume of 0.1 mL from each of the diluted sample was spread onto Columbia agar plates containing 5% (v/v) defibrinated sheep blood, without MeONQ. After incubation in a microaerophilic jar system (5% O_2_ and 10% CO_2_ in air) at 37°C for 72 h, the colonies formed on the Columbia agar plate were enumerated. Experiments were performed in triplicate.

### 2.8. Thermal Stability of MeONQ

The thermal stability of MeONQ on bactericidal activity against *H. pylori* ATCC 700824 was determined. MeONQ was prepared with DMSO at the concentration of 7.5 mg mL^−1^ and heated at 50, 70, and 100°C for 30 min; and 121°C for 15 min, then, immediately cooled in an ice bath. The treated MeONQ was subjected to the MIC testing. Unheated MeONQ was used as the control. Experiments were performed in triplicate.

### 2.9. MeONQ Levels in *I. balsamina*

 L. MeONQ levels in the different parts (pods, flowers, seeds, roots, stems and leaves) of *I. balsamina* L. were determined by a Hitachi HPLC system (Tokyo, Japan) consisting of a Model L-7100 pump equipped with a multi-solvent delivery system and an L-7455 photodiode array detector. The column was a Mightysil RP-18GP, 5 *μ*m, 4.6 mm in internal diameter (i.d.), and 250 mm in length (Kanto, Tokyo, Japan). The sample preparation was the same as the aforementioned, and 95% ethanol was used as the extraction solvent. The mobile phase was composed of a mixture of 2% acetic acid aqueous-methanol (1/1, v/v). An isocratic elution mode with 1.0 mL min^−1^ of flow rate was done. The UV absorbance was detected at 248 nm. The operating temperature was maintained at room temperature. Experiments were performed in triplicate.

### 2.10. Statistical Analysis

Data for time-kill assay, effect of pH for MeONQ, and MeONQ levels in *I. balsamina* L. were subjected to analysis of variance, and a *t*-test was used to identify significant differences among the means (*P* < .05).

## 3. Results

### 3.1. Isolation and Structure Elucidation of Anti-*H. pylori* Compounds

The pod extract of *I. balsamina* L. was subjected to silica gel column chromatography to produce compound (**1**). Compound (**1**) was identified as MeONQ by comparison of the spectroscopic data with those of the known compound [8, 16]. The *n*-hexane fraction of roots/stems/leaves extract of *I. balsamina* L. was subjected to silica gel column chromatography to give compound (**2**). Compound (**2**) was identified as stigmasta-7,22-diene-3*β*-ol (spinasterol) by comparison of the spectroscopic data with those of the known compound [8, 16, 20]. The chemical structures of MeONQ and spinasterol are shown in Figure 1.

#### 3.1.1. MeONQ (Compound 1)

Yellow powder; m.p. 182–184°C; UV (MeOH) *λ*_max_ 243, 248, 278, 338 nm; FTIR (KBr) *v*_max_ 1682 (C=O), 1648 (C=O), 1605 (C=C, Ar), 1244 (C–O) cm^−1^; for ^1^H and ^13^C NMR, see Table 1; EI-MS (+70 eV) m/z 188 [M]^+^ (31), 173 [M−CH_3_]^+^ (18), 158 [M−OCH_2_]^+^ (14), 102 (46), 89 (100). 

#### 3.1.2. Spinasterol (Compound 2)

White powder; m.p. 164–166°C; UV (EtOH) *λ*_max_ 203, 274 nm; FTIR (KBr) *v*_max_ 3448 (O–H), 2941 (C–H), 2869 (C–H), 1654 (C=C) cm^−1^; for ^1^H and ^13^C NMR, see Table 1; EI-MS (+70 eV) *m/z* 412 [M]^+^ (32), 397 [M−CH_3_]^+^ (13), 394 [M − H_2_O]^+^ (2), 379 [M−CH_3_−H_2_O]^+^ (2), 369 [M−C_3_H_7_]^+^ (18), 351 (7), 300 [M−C_3_H_12_O]^+^ (16), 271 [M−C_10_H_19_−2H]^+^ (100), 255 [M−C_10_H_19_−H_2_O]^+^ (50), 253 [M−C_10_H_19_−H_2_O−2H]^+^ (19), 246 (23), 231 [M−C_10_H_19_−C_3_H_6_]^+^ (18), 229 [M−0C_10_H_19_−C_3_H_8_]^+^ (22), 213 [M−0C_10_H_19_−C_3_H_6_−H_2_O]^+^ (19), 211 [M−C_10_H_19_−C_3_H_8_−H_2_O]^+^ (3).

### 3.2. MICs and MBCs for MeONQ and Spinasterol

The MICs and MBCs for MeONQ and spinasterol are listed in Table 2. Both MICs and MBCs for MeONQ were relatively low, in the ranges of 0.156–0.625 and 0.313–0.625 *μ*g mL^−1^, respectively. Compared with the positive controls, the MICs and MBCs of MeONQ were 25–400% of AMX (0.078–2.5 and 0.156–2.5 *μ*g mL^−1^ of MICs and MBCs, resp.); and much lower than those of MTZ (160–5120 *μ*g mL^−1^ of both MICs and MBCs). MeONQ demonstrated very good anti- and bactericidal *H. pylori* activity against CLR, MTZ, and LVX-resistant *H. pylori*, which was equivalent to that of AMX.

Both MICs and MBCs for spinasterol against six strains of CLR, MTZ and LVX-resistant *H. pylori* were 20–80 *μ*g mL^−1^ (Table 2). Compared with the positive controls, both the MICs and MBCs for spinasterol were higher than AMX and lower than MTZ.

### 3.3. Time-Kill Assay of MeONQ

**Figure 2:**
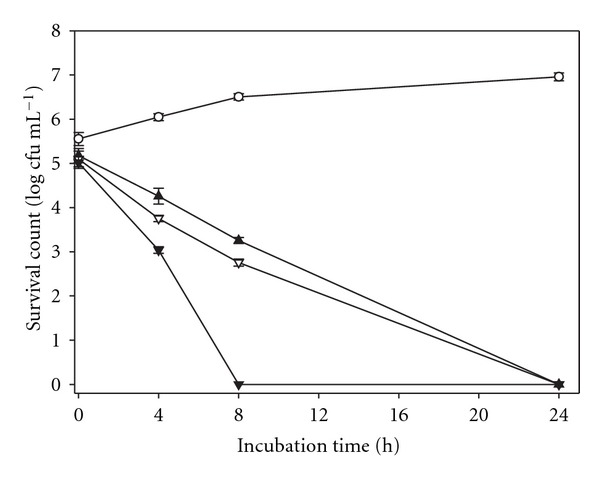
Time-kill assay for MeONQ against *H. pylori* ATCC 700824: control (open circles), 0.313 *μ*g mL^−1^ (filled triangles), 0.625 *μ*g mL^−1^ (inverted open triangles), 1.25 *μ*g mL^−1^ (inverted filled triangles). Data points presented are in mean ± standard deviation (SD) (*n* = 3).

As Figure 2 illustrates, three treatments (0.313, 0.625, and 1.25 *μ*g mL^−1^) initially reduced survival counts significantly by 0.37–0.53 log cfu mL^−1^ compared with the control. The 0.313 and 0.625 *μ*g mL^−1^ treatments (1 and 2 times MBC, resp.) reduced *H. pylori* survival counts from 5.10 ± 0.18–5.18 ± 0.16 log cfu mL^−1^ to 0 after 24 h cultivation; when the treated dose increased to 1.25 *μ*g mL^−1^ (four times MBC), the survival counts were reduced to 0 after 8 h. The bactericidal action between the three treated does was significantly different (*P* < .05), indicating that the bactericidal *H. pylori* ATCC 700824 action of MeONQ was dose-dependent.

### 3.4. Effect of pH of MeONQ

**Figure 3:**
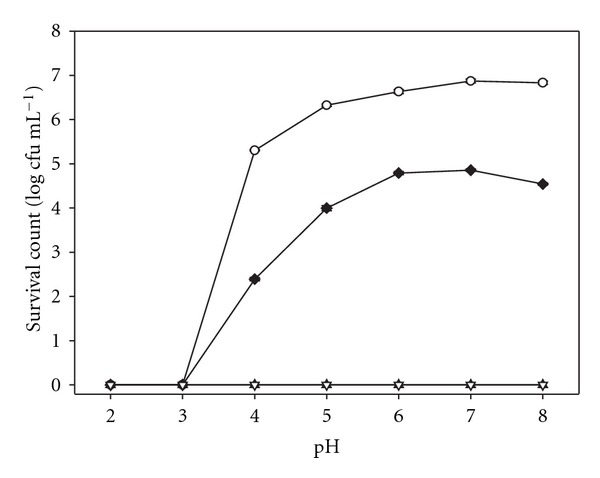
Effect of pH of MeONQ on the bactericidal activity against *H. pylori* ATCC 700824: control (open circles), 0.156 *μ*g mL^−1^ (filled diamonds), 0.313 *μ*g mL^−1^ (filled triangles), 0.625 *μ*g mL^−1^ (inverted open triangles). Data points presented are in mean ± SD (*n* = 3).

In Figure 3, at pH ≤ 3, the survival *H. pylori* ATCC 700824 counts were zero for all of the treatments including the control because those pH values stopped the bacterial growth. At pH 4–8, when the treated dose (0.156 *μ*g mL^−1^) was below the MBC (0.313 *μ*g mL^−1^), MeONQ reduced *H. pylori* ATCC 700824 survival counts by 1.84–2.91 cfu mL^−1^ after 24 h cultivation; while the treated doses were equal to or higher than the MBC, survival counts in all the treatments (pH 4–8) were reduced to zero after 24 h cultivation. The bactericidal *H. pylori* activity of MeONQ was not influenced by the environmental pH values (4–8).

### 3.5. Thermal Stability of MeONQ

After heat treatments (heating at 50, 70, and 100°C for 30 min; and 121°C for 15 min) of MeONQ, the MICs for the heat-treated MeONQ against *H. pylori* ATCC 700824 and KMUH 4917 (0.156 and 0.313 *μ*g mL^−1^, resp.) were the same as the untreated MeONQ, which indicated that MeONQ's anti-*H. pylori* activity was not altered even after strong heat treatment.

### 3.6. MeONQ Levels in the Parts of *I. balsamina*

 L. As shown in Table 3, the *I. balsamina* L. pods had the greatest amount of MeONQ (43.92 ± 1.56 mg g^−1^ db) which was 8–150 times that of the other parts. The MeONQ level in the flowers then followed at 5.45 ± 0.11 mg g^−1^ db. The levels in the parts of roots, stems, leaves and seeds were low (0.29 ± 0.00 to 0.56 ± 0.02 mg g^−1^ db) (*P* < .05).

## 4. Discussion

In this study, anti-*H. pylori* compounds, MeONQ and spinasterol, were isolated from *I. balsamina* L. Such anti- and bactericidal *H. pylori* activity had never been reported in those compounds. Especially, MeONQ exhibited relatively low MICs and MBCs against CLR, MTZ and LVX-resistant *H. pylori*. This activity was neither influenced by the environmental pH value nor strong heat treatment.

Numerous anti-*H. pylori* compounds including phenolics [23], flavonoids [24], triterpenoids [25], quinones [26] and others [27] were isolated from natural products. The MICs for those compounds varied from 1.3 to 200 *μ*g mL^−1^, however, none of them were lower than 1 *μ*g mL^−1^. Compared with our compounds, MICs for MeONQ against six *H. pylori* strains ranged from 0.156 to 0.625 *μ*g mL^−1^ which were much lower than those of the aforementioned compounds. In addition, those values were similar to AMX and much lower than MTZ (Table 2). MeONQ is the most effective bactericidal *H. pylori* natural compound which has thus far been reported.

**Figure 4:**
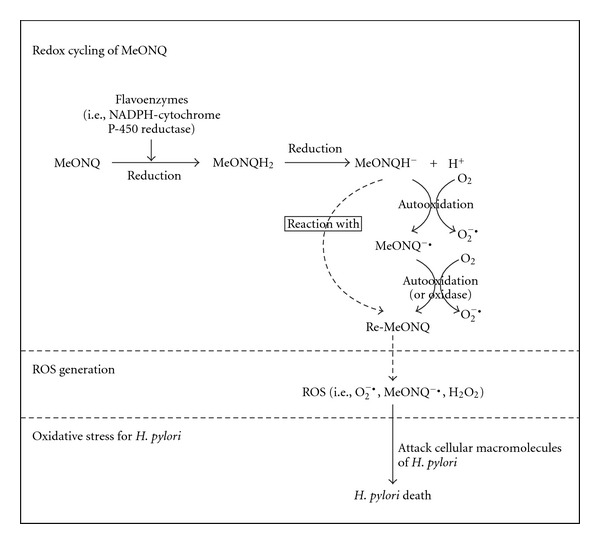
The hypothetical diagram for MeONQ that leads *H. pylori* death. MeONQ: 2-methoy-1,4-naphthoquinone; MeONQH_2_: hydroquinone derivative of MeONQ; MeONQH^−^: anion form of MeONQH_2_; MeONQ^−•^: semiquinone derivative of MeONQ; Re-MeONQ: regenerated MeONQ; ROS: reactive oxyge species.

We suppose that the mechanisms for the bactericidal *H. pylori* activity of MeONQ is due to the high redox potential of its hydroquinone form [28]. MeONQ is a quinone. Quinones are metabolized by flavoenzymes (such as NADPH-cytochrome P-450 reductase and xanthine oxidase) to form hydroquinones. The hydroquinones are quite unstable, which react with molecular oxygen to form semiquinones and superoxide anion radicals at physiological pH. Under aerobic conditions, the semiquinone free radicals are most likely to react with molecular oxygen to form superoxide anion radicals and quinones. The regenerated quinones react with anion form of hydroquinones resulting in large amounts of reactive oxygen species (ROS). These ROS can damage a number of cellular macromolecules and may lead to cell or microorganism death [28–30]. The hypothetical diagram is presented in Figure 4. 

Resistance to antibiotics are the greatest factor in *H. pylori* eradication failure, especially to MTZ and CLR. Resistance rates have increased annually over the last 15 years [4, 31]. For instance, the rates of 7.7% cross-resistance between MTZ and CLR in Poland and 21.7% between erythromycin and CLR in Ireland were reported [32, 33]. Thus, killing *H. pylori* is becoming more and more difficult. In our study, MeONQ not only exhibited relatively good anti- and bactericidal *H. pylori* activity but was also an effective bactericide against CLR, MTZ and LVX-resistant *H. pylori*.

AMX and CLR were not stable in aqueous solutions of pH lower than 4.0 and 5.0, respectively [34], and therefore degrade rapidly at normal gastric pH (1.0–2.0). From our results (Figure 3), both anti- and bactericidal *H. pylori* activity of MeONQ were not influenced by the environmental pH values (4, 8). Moreover, MeONQ exhibited very good thermal stability, even when heated at 121°C for 15 min. These physical properties highly increased MeONQ's efficiency in *H. pylori* eradication.

In this study, we first revealed that MeONQ abound in the *I. balsamina* L. pod as high as 43.92 ± 1.56 mg g^−1^ db (Table 3). As previously reported, MeONQ levels in the leaves, stems and flowers of *I. glandulifera* Royle were 1.98, 1.33, and 4.86 mg g^−1^ db, respectively [10]; and that of levels in 40 *I. balsamina* L. leaf samples were in a range of 0.3–1.9 mg g^−1^ db [8]. Our MeONQ level in the leaves of *I. balsamina* L. was similar to Panichayupakaranant et al. [8], lower than Lobstein et al. [10], and the level of that in the flowers was similar to Lobstein et al. [11].

## Funding

National Science Council, Taiwan, R.O.C. (grant no.: NSC 95-2313-B-005-057-MY3).

## Figures and Tables

**Table 1:** ^1^H and ^13^C NMR (600 and 150 MH_Z_, *δ* ppm, CDCl_3_) spectral data for compounds (**1**) and (**2**).

C/H	(**1**)		(**2**)	
	^1^H	^13^C NMR	^1^H	^13^C NMR
1		180.1		37.1
2		160.4		31.5
3	6.19 s	109.9	3.60 (^1^H, m)	71.0
4		184.9		38.0
5	8.13 dd (1.2, 7.2)	126.7		40.2
6	7.76 td (1.2, 7.2)	134.4		29.6
7	7.73 td (1.2, 7.2)	133.4	5.15 (1H, t, *J* = 12.0 H_Z_)	117.4
8	8.10 dd (1.2, 7.2)	126.2		139.5
9		131.0		49.4
10		132.0		34.2
11	3.92 s	56.4		21.5
12				39.4
13				43.3
14				55.1
15				23.0
16				28.5
17				55.8
18			0.55 (3H, s)	12.0
19			0.79 (3H, s)	13.0
20				40.8
21			1.02 (3H, d, *J* = 6.6 H_Z_)	21.1
22			5.15 (1H, dd, *J* = 9.0, 15.0 H_Z_)	138.2
23			5.02 (1H, dd, *J* = 8.7, 15.3 H_Z_)	129.4
24				51.2
25				31.9
26			0.80 (3H, d, *J* = 4.2 H_Z_)	19.0
27			0.85 (3H, d, *J* = 5.4 HZ)	21.4
28				25.4
29			0.80 (3H, t, *J* = 6.0 H_Z_)	12.3

**Table 2:** MICs and MBCs for MeONQ and spinasterol against *H. pylori.*

Strain	MIC (*μ*g mL^−1^)				MBC (*μ*g mL^−1^)			
	MeONQ	Spinasterol	AMX	MTZ	MeONQ	Spinasterol	AMX	MTZ
ATCC 700824	0.156	20	0.078	160	0.313	20	0.156	320
ATCC 43504	0.313	20	0.078	2560	0.625	40	0.156	2560
ATCC 43526	0.625	80	0.156	160	0.625	80	0.156	160
KMUH 4917	0.313	20	0.313	160	0.625	40	0.313	160
KMUH 4952	0.625	80	2.5	5120	0.625	80	2.5	5120
KMUH 4967	0.625	80	0.625	160	0.625	80	0.625	160

**Table 3:** MeONQ levels in the different parts of *I. balsamina* L.

Sample	MeONQ (mg g^−1^ db^a^)
Pod	43.92 ± 1.56a
Flower	5.45 ± 0.11b
Root	0.35 ± 0.01c
Stem	0.56 ± 0.02c
Leaf	0.29 ± 0.00c
Seed	0.37 ± 0.00c

Values presented are in mean ± SD (*n* = 3). Means followed by different letters are significantly different (*P* < .05).

^a^Dried base.
